# Multi-omics tumor profiling technologies to develop precision medicine in multiple myeloma

**DOI:** 10.37349/etat.2021.00034

**Published:** 2021-02-28

**Authors:** Sara Ovejero, Jerome Moreaux

**Affiliations:** 1 Department of Biological Hematology, CHU Montpellier, 34295 Montpellier, France; 2 Institute of Human Genetics, UMR 9002 CNRS-UM, 34000 Montpellier, France; 3 UFR Medicine, University of Montpellier, 34093 Montpellier, France; 4 Institut Universitaire de France (IUF), 75000 Paris, France; National Research Council (CNR), Italy

**Keywords:** Multiple myeloma, omics data, drugs, biomarkers, predictive models, personalized medicine

## Abstract

Multiple myeloma (MM), the second most common hematologic cancer, is caused by accumulation of aberrant plasma cells in the bone marrow. Its molecular causes are not fully understood and its great heterogeneity among patients complicates therapeutic decision-making. In the past decades, development of new therapies and drugs have significantly improved survival of MM patients. However, resistance to drugs and relapse remain the most common causes of mortality and are the major challenges to overcome. The advent of high throughput omics technologies capable of analyzing big amount of clinical and biological data has changed the way to diagnose and treat MM. Integration of omics data (gene mutations, gene expression, epigenetic information, and protein and metabolite levels) with clinical histories of thousands of patients allows to build scores to stratify the risk at diagnosis and predict the response to treatment, helping clinicians to make better educated decisions for each particular case. There is no doubt that the future of MM treatment relies on personalized therapies based on predictive models built from omics studies. This review summarizes the current treatments and the use of omics technologies in MM, and their importance in the implementation of personalized medicine.

## Introduction

Multiple myeloma (MM) is the second most common hematologic cancer after non-Hodgkin lymphoma and accounts for 1–2% of all cancers per year [1]. It progresses with severe associated morbidities like bone pain and fragility, anemia, renal failure, and increased risk of infections. MM is more prevalent in patients older than 65 years and, unfortunately, there is still not a definitive cure for it. Despite big recent efforts in genetic analysis and massive sequencing approaches, its causes remain elusive and, sooner or later, virtually all patients develop resistance to treatments and eventually relapse [2, 3].

At the cellular level, MM is characterized by accumulation in the bone marrow (BM) of aberrant clones of plasma cells (PCs). In physiological conditions, B cells that respond to an infection will differentiate to become PCs, which are the antibody-producing terminal differentiation state of the B lymphocyte lineage [4]. In MM, aberrant PC clones constantly secrete a monoclonal immunoglobulin (Ig). The most common MM clinical manifestations are referred to as CRAB (hypercalcemia, renal insufficiency, anemia, and bone lesions due to increased bone destruction and decreased osteogenesis), and the sustained Ig elevated levels eventually cause problems in several organs, which strongly impact quality of life of patients [5].

MM is a highly heterogeneous disease with huge genetic complexity. Patients with MM evolve from an asymptomatic stage: monoclonal gammopathy of undetermined significance (MGUS) characterized by low level (< 30 g/L) of monoclonal Ig in blood or urine, also known as M protein or paraprotein. It presents less than 10% of PC in the BM and absence of the organ damage typical of MM [6]. Suffering from MGUS increases the risk of developing MM at a rate of about 1% a year [7]. Smoldering MM (SMM) is distinguished from MGUS because of a significant increase in the risk of developing MM (10% per year in the first 5 years) [8]. As MGUS, SMM patients do not present CRAB symptoms [9]. At the cellular level, the PC clone at the origin of MGUS may slowly proliferate and eventually can give rise to more aggressive subclones that will cause SMM. Work in the past years has pointed to the clonal origin of MM and has established a correlation between clonal evolution and the transition between MGUS, SMM and MM. Indeed, MM derives from the branching evolution of different subclones, and changes in subclone prevalence control disease evolution, response to treatments, and eventual relapse [10–13].

At the molecular level, genetic abnormalities present in MGUS are the primary events involved in tumor initiation, whereas genetic lesions present in MM, but absent in MGUS, would be the secondary events involved in tumor progression. In order to define a genetic landscape of the disease, cytogenetic abnormalities related to MM were studied by conventional techniques, including fluorescence *in situ* hybridization (FISH) and karyotyping. FISH analyses of PC from BM aspirates classify MM regarding chromosome gain, loss, rearrangement and translocations (TCs). These cytogenetic abnormalities are studied at diagnosis and relate to patient prognosis. A primary TC classification of MM was built using expression of a TC target gene together with the expression of a D group cyclin [14, 15]. TC groups could also be detected using multiplexed real-time quantitative RT-PCR [16]. Recently, complementary tools, such as gene expression profiling (GEP) or whole-exome sequencing (WES) of large cohorts of patients have allowed the molecular classification of different groups of patients based on their genomic profiles and disease outcome [17, 18]. In the last decades, development of new drugs, more accurate risk stratification analyses and prediction of response to treatments have significantly improved the mean survival of MM patients. In this review, we summarize current treatments and focus on the use of omics technologies towards the implementation of personalized medicine in diagnosis and treatment of MM.

## Treatments in MM

Treatment of MM includes a series of chemotherapeutic molecules coupled or not with autologous stem cell transplantation (ASCT). The available therapies for MM and drug-specific resistance mechanisms have been recently and extensively reviewed elsewhere [2, 19, 20]. Yet, given the focus of many omics studies on predicting response to treatments and analyzing cellular and molecular changes due to drugs, we will provide a brief summary on the treatment currently used for MM (Table 1). MM new treatments under development or in clinical trials, including bi-specific T-cell engagers antibodies (BiTEs), bi-specific antibodies, antibodies drug conjugates (ADCs), and chimeric antigen receptor-T cell (CAR-T cell) therapy are out of the scope of this review and we direct the reader to other reviews focused on them [21–24].

**Table 1.** Agents approved for MM treatment

**Drug**	**Type**	**Target**	**Effect**	**Resistance**
Melphalan Melflufen Cyclophosphamide	Alkylating agents	DNA	DNA damage Impairment of DNA replication and transcription	Misregulation/mutation of DDR pathways [25–31] Increased antioxidant defenses [32] Import/export alteration [33] miRNA misregulation [34]
Bortezomib Calfilzomib Ixazomib	PI	PSMB5 (26S proteasome)	Cytotoxicity by accumulation of aberrant proteins	BM microenvironment Upregulation of aggresomal protein degradation pathways Increased autophagy Proteasome subunit mutations Cell cycle misregulation [19, 20, 35, 36]
Thalidomide Lenalidomide Pomalidomide	IMiDs	CRBN	Immune activation Impaired angiogenesis Impaired proliferation of tumor cells Apoptosis induction	Low CRBN expression or mutation of its downstream targets [37–39]
Dexamethasone Prednisolone Methylprednisolone	Corticosteroids	GC receptors	Gene expression regulation Anti-inflammatory Immunosuppressive	Excess of IL-6 Defects on GC receptors *FGFR3* overexpression [40–42]
Doxorubicin Pegilated liposomal doxorubicine (PDL)	Anthracyclines	DNA-Topo II	Impairment of DNA replication and transcription DSBs accumulation	DNA-Topo II mutations or misregulation [43–45] Efflux pumps overexpression [46]
Panobinostat	Histone deacetylase inhibitors	Histones	G1/S arrest Apoptosis Activation of tumor suppressor genes	Increased CXCR4, mTOR pathway activation, p21 up-regulation [47]
Daratumumab (DARA) Elotuzumab	Monoclonal Abs	CD38 CD319/ SLAMF7/CS1	ADCC, ADCP, CDC, immunomodulatory effects ADCC, NK-cell activation	Downregulation of the target Deregulation of ADCC, ADCP, CDC Stromal cell production of anti-apoptotic proteins PD1 and PD-L1 [48, 49]
Selinexor	Nuclear export inhibitors	XPO1	Nuclear export blockade Reduction of DDR proteins	(*In vitro*) alterations in signaling pathways downstream of XPO1 [50]

ADCC: antibody-dependent cell-mediated cytotoxicity; ADCP: antibody-dependent cellular phagocytosis; CDC: complement-dependent cytotoxicity

### Alkylating agents

Melphalan is a nitrogen mustard that alkylates guanine bases in DNA, causing interstrand crosslinks (ICLs) that impair DNA replication and transcription, which results in cytotoxicity (reviewed in [51–53]). A derived compound named melflufen (melphalan flufenamide ethyl ester) has been proposed as an alternative to overcome melphalan resistance without differential toxicity [54, 55].

At high doses, cyclophosphamide metabolization by cells with low levels of aldehyde dehydrogenases produces the alkylating agent phosphoramide mustard, which causes irreversible DNA ICLs as melphalan does. Cyclophosphamide also decreases the immune response and it has been proposed that in low doses it can be used as an immunomodulatory drug (IMiD) in MM (reviewed in [56, 57]).

Historically, alkylating agents, mainly melphalan, were the standard-of-care for MM patients. Although their use is still widespread, frequent relapse of melphalan-treated patients has led to its use in combination with other drugs described in the following sections.

### Proteasome inhibitors (PIs)

Tumor cells often accumulate misfolded or aberrant proteins, which leads to increase proteasome levels to eliminate these proteins and cope with their potential toxicity. Hence, proteasomes are an attractive therapeutic target to treat some types of cancer [58]. Such is the case of MM, in which malignant PCs produce high amounts of monoclonal Ig and, therefore, are very sensitive to proteasome inhibition [59].

Bortezomib temporarily inhibits the activity of the proteasome subunit beta type-5 (PSMB5) subunit of the 26S proteasome, which impairs degradation of pro-apoptotic proteins and activation of the anti-apoptotic nuclear factor kappa B (NF-κB) pathway, among others [59]. Other PIs with less secondary effects have been developed. Carfilzomib, the second-in-class PI [60], binds to and irreversibly inhibits the proteasome, even in bortezomib-resistant cells, and has shown improved safety compared to bortezomib [61].

The first orally available PI, ixazomib was more recently developed. As bortezomib, it reversibly inhibits the PSMB5 proteasome subunit [62].

### IMiDs

The IMiDs induce immune activation and impair angiogenesis and proliferation of tumor cells through several targets. The first-in-class IMiD was thalidomide, that exerts anti-angiogenic and anti-tumoral activity. Thalidomide disrupts MM cells interaction with the BM microenvironment by inhibiting the production of cytokines that are essential for cell growth and survival [63, 64]. Its primary target is the E3 ubiquitin ligase complex cereblon (CRBN), whose inhibition leads to accumulation of proteins causing cytotoxicity [65] and down-regulates the transcription factors Ikaros (*IKZF1*) and Aiolos (*IKZF3*) leading to *IRF4* and MYC MM on cogene downregulation [37, 66].

Two analogs of thalidomide have been developed, lenalidomide and pomalidomide, which have shown more potent anti-MM, anti-inflammatory, and immunomodulatory effects than thalidomide [67], and in addition, induce cell arrest and apoptosis [68–71].

### Corticosteroids

Glucocorticoids (GCs) are a type of corticosteroids used to treat cancer for over 50 years. Dexamethasone, prednisolone, and methylprednisolone are the GCs used in MM. They bind GC receptors in the cytosol and induce their nuclear relocation. Once in the nucleus, they bind the GC response elements to regulate gene expression, either activating (Annexin I, MAPK) or inhibiting it (NF-κB, AP-1) [72, 73]. GC promotes anti-inflammatory and immunosuppressive activities. They induce apoptosis by affecting several pathways, poly (ADP-ribose) polymerase (PARP) cleavage and caspase 3 activation [74–76]. Due to dexamethasone toxicity, the use of prednisone instead has been proposed, especially for older patients [77]. A detailed review on GC use in MM has been recently published elsewhere [76].

### Anthracyclines

Anthracyclines are a class of drugs extracted from *Streptomyces* bacteria. Doxorubicin (also known as adriamycin) is the most relevant anthracycline in the treatment of MM. It acts as a DNA intercalating agent, inhibiting DNA replication and transcription. Doxorubicin forms a stable DNA-topoisomerase II-anthracycline complex that impairs topoisomerase II-mediated DNA religation, leading to double-strand breaks (DSBs) accumulation and cell death [78, 79].

However, doxorubicin high cardiotoxicity has limited its use. In the 90s, it was reformulated in liposomes [pegylated liposomal doxorubicine (PLD)] to improve its delivery to cells and reduce its toxicity [80].

### Histone deacetylase inhibitors (HDACis)

Acetylation and deacetylation of lysine residues in the N-ter of histones that form the nucleosome regulate gene expression. These post-translational modifications are epigenetic markers catalyzed by histone acetyltransferases (HAT) and histone deacetylases (HDAC). Histone acetylation relaxes chromatin structure and increases transcription levels while deacetylation has the opposite effect. The expression of critical genes related to cancer, like *TP53* or *BCL-2*, is regulated by acetylation [81]. Several HDACis have been evaluated in clinical trials: vorinostat [82, 83], ricolinostat [84], romidepsin [85], ACY-241 [86], and panobinostat; of them, only panobinostat has been approved for the treatment of MM [87–89].

Panobinostat is a pan-HDACi that reduces MM cell proliferation, arresting cell cycle at G1/S by affecting the p53 pathway, and induces apoptosis at low doses [87]. Panobinostat alone does not have a significant effect on MM; therefore, it is used in combination with other drugs [87, 90–92]. For a recent review on panobinostat use and clinical trials in MM see [89].

### Monoclonal antibodies (mAbs)

Immunotherapy uses mAbs that recognize highly specific antigens on the surface of MM cells leading to cell death. mAbs act through different mechanisms, such as direct cytotoxicity or boosting immune response against malignant cells. In the case of MM, mAbs have been developed to target two proteins: CD38 and CD319. On one hand, CD38 is a transmembrane glycoprotein with functions in signal transduction, calcium signaling and cell adhesion; it is normally expressed by plasmablasts and PCs, and overexpressed by MM cells, which makes it a selective target for the treatment of the disease. On the other hand, CD319 [also known as signaling lymphocytic activation molecule family member 7/CD2 subset-1 (SLAMF7/CS1)] is a stable surface marker of hematopoietic cells, in particular of NK cells, and normal and malignant PCs, which promotes cell growth and survival, and is involved in the interaction of MM cells with BM microenvironment [93, 94].

Several anti-CD38 mAbs have been developed: daratumumab (DARA), isatuximab, MOR202 and TAK-079; of them, only DARA has been approved to treat MM [95–97]. DARA induces immune-mediated cytotoxicity of CD38^+^ cells, and has been suggested to exert immunomodulatory activity, improving clinical responses in heavily pretreated patients [49, 98–100]. Currently, DARA is being studied as monotherapy or in combination with other drugs. The first clinical trials for DARA have provided encouraging results about its use alone [101] or together with other drugs [102–104].

Elotuzumab is an anti-CD319 mAb approved for the treatment of relapsed MM patients. It causes myeloma cell death by activating NK cells or through antibody-dependent cellular toxicity [105]. Elotuzumab has no significant activity when used as monotherapy [106], but it has synergistic anti-myeloma activity in combination with other drugs [107, 108].

The use of mAbs in MM has been recently reviewed elsewhere [49, 109].

### Selective inhibitors of nuclear export (SINEs)

Multi-drug resistance (MDR) is a common phenomenon in MM patients. MDR is mainly attributed to the overexpression of the ABC superfamily of ATP-dependent efflux transporters, in particular of P-glycoprotein (P-gp) and multidrug resistance-associated protein 1 (MRP1), encoded by the *ABCB1* and *ABCC1* genes respectively, and also lung resistance protein (LRP). These transporters can export drugs out of the cell, reducing their intracellular concentrations and diminishing their effect [110].

The cell surface transporter P-gp was identified a long time ago as responsible for drug pump out of the cell. It was associated with resistance to anthracyclines or taxanes, and later to alkylating agents or IMiDs. Moreover, it has been shown that many chemotherapeutic drugs, such as carfilzomib or doxorubicin, induce an up-regulation of P-gp expression [42].

In 2019, the first inhibitor of nuclear export, Selinexor, has been approved (reviewed in [111]). Selinexor binds to exportin 1 (XPO1), a transporter that mediates nuclear export of RNAs and proteins involved in tumor suppression, cell cycle, growth and apoptosis, and has been involved in hematologic malignancies [112]. Of note, XPO1 overexpression has been reported in MM associated to poor prognosis [113].

The use of selinexor in MM has been recently reviewed elsewhere [114].

### High-dose chemotherapy rescued by ASCT

ASCT was introduced in the 80s as a therapy in MM [115, 116]. Currently, ASCT is a standard-of-care for young and some eligible elderly patients at first diagnosis. Nevertheless, ASCT is not exclusive for newly diagnosed patients and can be indicated at relapse or progression of the disease [117].

Briefly, CD34^+^ stem cells from the patient are collected from blood, an induction treatment with 2–3 drugs is followed by high-dose melphalan or sometimes radiation to deplete the BM where malignant PCs are, and afterwards the CD34^+^ stem cells are infused back into the patient’s blood [118–120]. However, despite significant event-free survival observed post-ASCT, it does not rule out the possibility of relapses, which remain very frequent [121].

For more insight into ASCT protocols and perspectives, see [121] and [122].

## Omics in MM

Omics are novel, unbiased, comprehensive approaches for quantification and characterization of biological molecules, such as nucleic acids, proteins, lipids, and metabolites. The development of high-throughput omics technologies has brought substantial improvement in the management of patients with all types of hematologic and solid cancers.

The complete and quick information obtained by omics analyses is especially relevant in the case of MM, which is a molecularly very complex and rapidly evolving cancer. Current standard-of-care treatments have substantially improved the prognosis of patients, which can expect a long event-free progression of the disease and significant reduction of its associated morbidities, and even complete remission in some cases [123, 124]. However, MM is characterized by an extremely high incidence of patients who initially respond positively to treatment, but eventually develop resistance to one or multiple drugs [125].

In recent decades, much effort has been devoted to identifying molecular signatures, known as biomarkers, that can predict risk at diagnosis, response to conventional drugs, and the risk of relapse. In addition, numerous clinical trials have studied therapeutic combinations of standard-of-care drugs and new therapeutic molecules to overcome drug-resistance. However, the rationale to use these strategies remains incomplete. The combination of multiple drugs with different targets and pleiotropic downstream effects exponentially increases the complexity of the response and refractory mechanisms of MM, and complicates their study.

The implementation of more sensitive and affordable omics approaches has uncovered that classical MM genetic aberrations [TCs, copy number aberration (CNA)] are not the only important biomarkers in MM. High-throughput analysis of other molecular profiles has equally provided critical information to develop scores that help to stratify the risk and predict the benefit and response to therapies. In the following sections, we will provide a global vision on MM omics studies and their contribution to personalized drug therapy implementation.

### Genomics

For a long time, the classification of newly diagnosed MM (NDMM) patients and subsequent treatment decisions were based on conventional use of cytogenetics, FISH, and single nucleotide polymorphism (SNP) arrays [126]. Identification of chromosome TCs, deletions and amplifications allowed the initial stratification as low- or high-risk MM.

There is not a single common genetic driving event of MM, rather many genetic alterations that differ from patient to patient. A commonly accepted classification defines 7 big molecular groups associated to different risks [17] and some often-mutated genes with prognostic value have been identified [13, 127]. Given this genetic complexity, the limited information provided by karyotype, FISH and SNP microarrays is not sensitive enough to identify the genetic signatures required to guide treatment decisions. Highly performant next generation sequencing (NGS) techniques allow the accurate sequencing of a complete genome within a short time. In MM patients, this capacity to fully sequence the genome is particularly interesting, since the natural course of the disease involves the fast accumulation of new mutations that may lead to relapse or refractory MM (RRMM). Custom capture NGS panels have been developed to identify rearrangements at the *IGH* locus, CNAs, and frequently mutated genes, with high sensitivity and specificity [128, 129]. This kind of approaches can capture somatic point mutations and provide more precise information than conventional techniques, which makes them good candidates to eventually replace them in clinical practice. Several consortium or groups have developed large datasets from multiple MM patient cohorts (Table 2) representing an extremely useful resource widely used in genomic studies to tackle questions about MM physiopathology and response to different treatments. These studies paved the way for the advent of genomics based-precision medicine in MM.

**Table 2.** Datasets from MM patients used in genomics studies presented in this review

**Dataset**	**Technique**	**Number of patients**	**GEO series accession number**
HOVON-65/GMMG-HD4	Affymetrix HG U133 plus 2.0 platform	320	GSE19784
UAMS-TT2 UAMS-TT3	Affymetrix HG U133 plus 2.0 platform	340 214	GSE24080
MRC-IX	Affymetrix HG U133 plus 2.0 platform Gene Chip Mapping 500K Array set Genome variation profiling by SNP array SNP genotyping by SNP array	258 114	GSE15695
APEX/SUMMIT/CREST	Affymetrix U133 A/B platform	669	GSE9782
IFM-G	Affymetrix HG U133 plus 2.0 platform	182	GSE7039
Mayo Clinic cohort	Affymetrix U133A platform	162	GSE6477
CoMMpass	WES: Illumina TruSeq Exome Enrichment WES: Agilent SureSelect Human All Exon + untranslated regions (UTRs) Library RNA-Seq: Illumina truSeq Stranded mRNA	1,150	dbGaP phs000748.v7.p4
HM	Affymetrix HG U133 plus 2.0 platform	206	Array Express public database (E-MTAB-372)
GIMEMAMMY-3006		118	GSE68871

GEO: Gene Expression Omnibus; HOVON65/GMMG-HD4: Dutch-Belgium Hemato-Oncology Group and German-speaking Myeloma Multicenter Group; UAMS-TT2/UAMS-TT3: University of Arkansas for Medical Sciences-Total Therapy 2/3; MRC-IX: Medical Research Council-IX; APEX: Assessment of Proteasome Inhibition for Extending Remissions; IFM-G: Intergroupe Francophone du Myelome; CoMMpass: the Relating Clinical Outcomes in MM to Personal Assessment of Genetic Profile Study; HM: Heidelberg-Montpellier; UTRs: untranslated regions

The importance of genomics studies in MM has been recently reviewed elsewhere [130].

#### MM physiopathology and risk stratification

Several genome-wide association studies (GWAS) have identified 23 susceptibility loci for MM [131–135]. The most recent GWAS meta-analysis also integrated information from gene expression, epigenetic profiling and *in situ* Hi-C data to identify the key altered pathways in MM, including disruption of developmental transcriptional regulators, autophagy and apoptosis [136].

In the search for driver mutations in MM, WES studies have identified frequently mutated genes, including *TP53*, *IRF4*, *KRAS*, *NRAS*, and NF-κB pathway genes [136–138]. High throughput analyses of gene mutations provide large amount of data that require downstream functional studies to determine which mutations have a real phenotypic impact. In addition, it is important to keep in mind that mutations that occur in non-coding regulatory regions can also impact gene expression.

In this regard, the search for mutations has been extended to *cis*-regulatory elements and promoters. Non-coding region whole genome sequencing (WGS) data from naïve B-cells from 765 MM patients analyzed in the Relating Clinical Outcomes in MM to Personal Assessment of Genetic Profile Study (CoMMpass) dataset found recurrently mutated promoters of genes associated to cell adhesion, inflammatory response, NIK-NF-κB, B-cell activation, and B-cell differentiation pathways. Of note, some genes central to PC differentiation, such as *IRF4*, *PRDM1*, *BCL6* and *PAX5*, were found to be affected by coding and non-coding mutations. Data from non-coding regions were integrated with information from coding regions, structural variants and mutational signatures to depict a comprehensive mutational landscape of MM [139]. Further functional characterization of the novel mutated genes would be useful to better understand the oncogenic pathways driving MM and find potential new druggable targets.

In the past years, genomics approaches have also been used to study clonality of MM and its role in progression from MGUS/SMM to MM [140–142]. WGS was used to study the progression of MM from diagnosis over a 5-year period in a high-risk t(4;14) patient. This work pointed at an initial heterogeneity of the tumor that shifted to tumor clones and acquisition of mutations over time [143], a phenomenon that had been previously described in less detail at the level of chromosome aberrations and TCs using FISH [140], SNP arrays [144], and array-based comparative genomic hybridization (CGH) [145]. Interestingly, WES data also suggested that tumor PC independence from BM microenvironment is due to accumulation of changes in the genome [136]. WGS and WES studies reported that, in general, the number of mutations increases from MGUS to MM and that the potentially predominant MM clone is already present at the SMM stage [141]. An increase in CNAs from MGUS to SMM to MM detected by SNP-arrays has also been reported [144]. A more recent study confirmed that aberrant PC subclones are already present in MGUS and SMM, but reported that mutational landscape changes with the progression of the disease rather than increasing the total number of mutations [142]. Further studies are necessary to better understand how selection pressure driven by the competition for BM niches and evasion of immune responses shapes subclonal evolution during premalignant to MM stages. Longitudinal study of MM patients by GEP, high resolution copy number arrays and WES underlined the importance of acquired bi-allelic inactivation of tumor suppressor genes in association with high disease aggressivity [146].

The study of omics at the single cell level is commonly known as “single-cell analysis” and it is a growing research field. Single-cell analysis allows the identification of differences between cells within populations that would go unnoticed in bulk population analyses. In 2012, a single-cell genomics study allowed the precise quantification of the percentage of cells in the population that carried a particular mutation or combination of mutations (for example *ATM*, *ATM-FSIP2*, or *ATM-CLTC-GLMN* [147]). Another work combined single cell genetics and WES to study parallel and branching evolution patterns of MM clones [148]. Interestingly, single-cell analysis of circulating tumor cells from peripheral blood has been suggested as a minimally invasive and sensitive alternative to BM biopsies to study mutations and clonal evolution of MM [149]. There is no doubt that the use of more performant single-cell approaches in the upcoming years will help to characterize molecular alterations at the origin of MM cells, clonal evolution, and drug resistance.

#### Identification of genetic biomarkers for MM risk stratification

In 2011, the first approach to sequence a cohort of MM genomes was based on WGS and WES of 38 patients. Analysis of MM genomes and comparison to normal ones found mutations in genes involved in several biological processes (protein translation, histone methylation, and blood coagulation) that were not related before to MM [150].

This study paved the way to the identification of genetic biomarkers that could help to predict risk level at diagnosis. One of the most commonly mutated pathways in MM is NF-κB pathway [151]. Genomic studies using SNP arrays, high-density CGH arrays, WGS and WES on several large patient cohorts identified a set of frequently mutated genes in MM, some of them belonging to NF-κB pathway: *TP53*, *KRAS*, *NRAS*, *BRAF*, *MAX*, *FAM46C*, *DIS3*, *IRF4*, *HIST1H1E*, *EGR1*, *LTB*, *FGFR3*, *TRAF3*, *CYLD*, and *RB1*. Mutations on some of them may be both MM initiator and disease potentiator events, and have prognostic value [10, 136–138, 147, 150, 152–154].

Interestingly, a model that identifies patients with long survival has been developed integrating deep WGS and clinical data. All NDMM patients were treated with lenalidomide, bortezomib, and dexamethasone alone or plus ASCT. A subgroup of patients with low DNA damage and mutational load had prolonged survival compared to the rest of the cohort [155]. This study built the genome scar score (GSS) to identify patients with potential longer survival which may benefit from longer and less toxic therapies. Moreover, identification of such patients could be of importance for clinical trial design too, for example when studying consolidation therapies or reducing treatment toxicity.

Combination of SNP-based gene mapping and global GEP identified 170 genes with homozygous deletions relevant to MM physiopathology and prognosis. An initial 97-cell death gene signature was developed and subsequently simplified to a 6-gene signature (known as *MRCIX6*) that can predict poor-prognosis [156].

Other predictive models based on analysis of gene mutations and/or GEP have been published (Table 3). The incorporation of these scores at diagnostic as well as disease monitoring would be instrumental to improve therapeutic decisions to increase survival and achieve long-lasting remission.

**Table 3.** Prognostic scores for MM based on mutational status and/or GEP

**Score**	**Genes**	**Datasets**	**Reference**
Proliferation index	11 genes: *TOP2A*, *BIRC5*, *CCNB2*, *NEK2*, *ANAPC7*, *STK6*, *BUB1*, *CDC2*, *C10orf3*, *ASPM*, and *CDCA1*	UAMS-TT2 UAMS-TT3	[17]
UAMS70/UAMS17	70/17 genes High risk: over expression of chromosome 1q genes and reduced expression of 1p genes	UAMS	[157]
UAMS80	80 genes	UAMS-TT2 UAMS-TT3	[158]
GSS	Good-risk: allele-specific CAN genomic markers	IFM/DFCI2009 study CoMMpass	[155]
HM19	19 genes (15 risk and 4 protective)	HM UAMS-TT2	[159]
CTA (cancer testis antigen)	87 genes Most relevant genes: *MAGEC1*, *MAGEB2*, *SSX1*, *MAGEA6*, *CDCA1*, *MAGEA9*, *CTAG2*	HOVON65/GMMG-HD4 APEX/SUMMIT/CREST	[160]
CI	Centrin, pericentrin, γ-tubulin	UAMS-TT2 Bortezomib trial Mayo Clinic cohort HMCLs	[161, 162]
IFM15	15 genes (cell cycle genes)	IFM-G	[163]
MRCIX6 (aka HZCDC)	*BUB1B vs. HDAC3* *CDC2 vs. FIS1 RAD21 vs. ITM2B*	Medical Research Council-IX (MRC-IX)	[156]
GPI50 PI	50 genes Poor prognosis: gain of 1q21 or deletion of 13q14.3 Good prognosis: gain of chromosome 9, 15 or 19	HM1 (E-MTAB-316) HM2 (E-MTAB-317) E-GEOD-2658 GSE4581	[164]
EMC-92 (SKY92)	92 genes Most relevant genes: *FGFR3* and *BIRC5*	HOVON-65/GMMG-HD4 TT2, TT3, MRC IX, APEX	[165–167]
HM-metascore	Algorithmic integration of International Stating System (ISS), cytogenetics, gene-expression, event-free survival (EFS), overall survival (OS), proliferation index, target gene expression of aurora kinase A, *FGFR3*, *IGF1R*	HM (E-MTAB-372) UAMS-TT2 MMRC (Multiple Myeloma Research Consortium data)	[168]
8-gene signature	*ATF2*, *CCND2*, *CFLAR*, *DDX17*, *HSPA1A*, *RIT1*, *RNF148*, *WHSC1*	GSE16791	[169]
Spike band score	53 (35 bad prognosis and 18 good prognosis)	HM (E-MTAB-362) UAMS-TT2 (GSE2658) HMCLs (E-TABM-937 and E-TABM-1088)	[170]
HMCL7/HMCL6	7 bad prognostic genes: *TEAD1*, *CLEC11A*, *LRP12*, *MMSET*, *FGFR3*, *NUDT11*, and *KIAA1671* 6 genes: *FSTL5*, *GAGE1*, *GAGE12*, *BCHE*, *HOOK3*, and *LOC283352*	HMCLs (E-TABM-937 and E-TABM-1088)	[171]
CINGECS	160 genes	GSE26849 GSE26760 GSE2658 UAMS GSE9782 APEX GSE19784 HOVON	[172]

CAT: cancer testis antigen

#### Identification of genetic biomarkers for treatment response

SNP arrays and WES on serial samples have shown that malignant MM PC subclones may follow different evolution patterns, including linear and branching evolution, and differential subclonal responses to treatment, which favors positive selection of resistant subclone(s) [136, 173, 174]. The analysis of the most common mutations suggested that targeted treatments can have suboptimal efficacy when certain mutations are present only in a sensitive subclone, and not in the whole malignant PC population, in which the other subclones not harboring the mutations would be resistant [137]. Targeted sequencing of PCs from BM of 43 MM patients at diagnosis identified the most frequently mutated genes (*KRAS*, *NRAS*, *DIS3*, *BRAF*, and *LRP1B*), confirming the results from previous reports. The same patients were analyzed at relapse after treatment with bortezomib-thalidomide-dexamethasone. Interestingly, no specific mutation at relapse was identified, suggesting that treatment effect on clonal selection is nonspecific [12]. We recently used WES to further characterize the mutational landscape of human MM cell lines (HMCLs) and their response to ten conventional drugs. We identified a high confidence list of 236 genes, common MM driver mutations (i.e. *TP53*, *KRAS*, *NRAS*, *ATM* and *CCND1*) and novel mutated genes belonging to JAK-STAT, PI(3)K-AKT, DNA repair and chromatin modifier pathways, with a focus on their correlation to drug response [175]. This information will help to design personalized treatments based on each patient’s clonal genetic background.

An alternative technique to classic WGS, low-depth whole genome sequencing (LD-WGS), has been recently evaluated in comparison to FISH for characterization of MM samples. LD-WGS would be more cost-effective than FISH, showing better sensitivity and resolution for CNAs detection [176]. Custom target pulldown (TPD) panels have been designed to detect gene mutations, CNAs, and TCs commonly found in MM. Design of TPD panels is based and depends on massive sequencing approaches to choose the most significant and relevant genes. TPD was used for the genomic characterization of 418 NDMM patients and PI- and IMiDs-treated patients with long follow-up, correlating the results with prognosis and classification into risk groups. This study showed that gene mutations have less predictive value than CNAs and TCs in the prognosis definition [18]. The MM-specific mutation panel (M^3^P) contains the most frequently mutated genes and incorporates a selection of clinically relevant ones because of their association to drug resistance [177–179]. Compared to FISH and WES/WGS, they provide a cheaper and faster alternative to characterize the mutational landscape of MM patients prior to therapy decisions, which makes them a suitable future option for standard-of-care protocols. Incorporation to clinical routine of these and future faster, cheaper and more performant technologies will be crucial for a better treatment of MM patients.

Minimal residual disease (MRD) designates the small number of BM cancer cells that remain during or after treatment when the patient is in remission. MRD is defined as negative when there is less than 1 malignant PC per million of analyzed BM cells [180]. MRD is a critical factor that could be systematically monitored all along the treatment to predict patient outcome [181]. MRD was previously assessed by multiparametric flow cytometry, that is the base for the next-generation flow (NGF) method [182, 183]. Mainly, three new methods assess the detection of MRD:NGF, allele-specific oligonucleotide PCR, and NGS [184, 185]. The three methods are highly sensitive, and have pros and cons based on the amount of starting material they require, reproducibility, time and economical cost, and complexity of data analysis [184, 186]. Recent data have demonstrated the superior performance of NSG in detection of MRD in MM [180], but it is also more expensive, time consuming, and difficult to analyze [186]. According to the differences, these methodologies could present complementary interest in clinical routine.

In summary, genomic approaches are providing important insight into MM molecular mechanisms, which were impossible to characterize with classical cytogenetic techniques. Genomic analysis of patient cohorts and *in vitro* models also allows to build predictive models to stratify the risk at diagnosis. If implemented in a clinical setting to characterize the initial mutation burden, these scores would be extremely helpful to design a tailored treatment for each patient. Moreover, determining changes in the mutational landscape of the tumor upon treatment would allow to readapt therapy based on subclonal evolution, which in turn would prolong patient survival and increase the chances of remission.

### Transcriptomics

The level of expression of potential prognostic genes is as relevant as DNA alterations themselves to understand MM, assessing risk at diagnosis, and designing targeted therapies. In the past decades, the revolution in the sensitivity of transcriptomics approaches has revealed new molecular mechanisms and provided a deeper insight into others already not fully understood.

Combination of WES and RNA-seq determined that the majority of mutations found in a group of 10 MM patients occurred within low- or non-expressed genes, which suggests that they may have low or no functional relevance [187]. This idea highlights the added value of combining DNA sequencing and gene expression data to identify the most relevant genes in MM and the sometimes-low correlation between genomics and transcriptomics data.

RNA-sequencing has also been used to create the first map of gene fusions in MM. Data from 71 HMCLs and 255 NDMM patients showed that patients have on average 5.5 expressed fusion genes, most of them affecting kappa/lambda light chains and *IGH* genes, and that the number of fusions increases with age. Four hundred and ninety five genes were found to be involved in at least one fusion, specially genes from chromosome 19. The most affected pathways were TNFα signaling via NF-κB, p53 and apoptosis pathways. Of the 36 genes most recurrently involved infusions, 2 were associated to lower progression-free survival (*CSNK1G2* and *CCND1*) and 2 to shorter overall survival (*MMSET* and *BCL2L11*), and patients with more than 16 fusions had poorer prognosis [188].

MM dependence on NF-κB pathway was described a long time ago [189], but the underlying molecular mechanisms were poorly known. GEP identified increased expression of NF-κB target genes as a common feature of MM primary cells and HMCLs, and several genetic and epigenetic mechanisms responsible for this NF-κB upregulation that could be interesting therapeutic targets [151]. Major TCs involving the *IGH* locus, such as t(4;14) or t(11;14), are well known and extensively characterized in MM. However, the frequency of less common TCs is poorly studied. Targeted RNA-seq of 21 patients allowed the identification of 2 novel fusions (*HGF*/*CACNA2D1* and *SMC3*/*MXI1*) whose biological and clinical relevance for MM physiopathology has not yet been determined [190]. Additionally, 94 genes at 8 regions that were not previously associated to MM risk have been identified by integrating genome-wide and transcriptome-wide association studies [191]. As already mentioned, personalized clinical management of MM requires predictive scores to assess risk at diagnosis and anticipate responses to drugs. A number of prognostic gene expression signatures have been identified in MM [157, 159, 161, 163, 165, 170–172] (Table 3) from direct comparison of patients with survival or from important biological processes of prognostic importance. A high-risk MM score was defined with70 deregulated genes [“University of Arkansas for Medical Sciences-Total Therapy 70 (UAMS70)” score], most of them mapping to chromosome 1. Interestingly, a model including 17 of those genes (*KIF14*, *SLC19A1*, *CKS1B*, *YWHAZ*, *MPHOSPH1*, *TMPO*, *NADK*, *LARS2*, *TBRG4*, *AIM2*, *ASPM*, *AHCYL1*, *CTBS*, *MCLC*, *LTBP1*) had similar predictive capacity as the 70-gene model [157]. The same group reported a GEP study of PC 48 h after thalidomide and dexamethasone, or bortezomib treatment, developing the “UAMS80” score, which includes 80 genes predicting survival [158]. Of note, 3 genes were common to UAMS70 and UAMS80: *PMSD4*, *BIRC5* and *KIAA1754*. Similarly, GEP analysis found that high-risk MM patients are characterized by overexpression of cell cycle genes (cell cycle progression, mitosis, spindle assembly checkpoint, DNA replication and repair). These data were used to build a survival model including 15 genes belonging to these molecular pathways, such as *MAD2L1*, *PARP1*, *BUB1B*, and *ZWINT*, to stratify low- and high-risk patients [163]. In agreement with these results, another GEP study determined that proliferation is a central prognostic factor in MM [164]. Similarly, we used Affymetrix microarrays data to develop and validate a score based on highly transcribed genes in MM that demonstrates prognostic value in NDMM [170]. More recently, Chng and collaborators [192] analyzed the interest to combine different already published gene expression risk-signatures to improve prognostic stratification in MM. Interestingly, the comparison of chromosome instability genome event count signature (CINGECS), CI, EMV92, HMCL7, HZCDC, IFM15, PI, UAMS70 and UAMS80 (Table 3) and analysis of the performance of different score combinations determined that EMC9 + HZCDC was the top-performing prognostic signature combination [192]. This conclusion indicates that integrating several predictive scores may be the best approach to optimal risk estimation.

Single-cell RNA-seq of PCs from patients has identified some of the most affected pathways (i.e. cell metabolism and protein homeostasis pathways) during MGUS to MM progression, providing a new signature for prognosis and therapy stratification [193]. Targeted transcriptome single-cell analysis was used to develop a bioinformatic tool to analyze gene expression changes induced by PI at the subclonal level that could contribute to improve therapeutic choices [194]. Of note, we demonstrated that a collection of 40 HMCLs derived from patients recapitulated the molecular diversity of *in vivo* MM. This analysis provided a genetic signature for stratification of patient risk and proved that this HMCLs collection is an invaluable *in vitro* model for the study of MM and the screen of potential new therapies [171]. A recent study described single cell dissection of malignant PC transcriptome in symptomatic and asymptomatic MM patients [195]. They described intrasubject transcriptional heterogeneity and revealed that circulating MM cells reflect the molecular heterogeneity in MM cells of BM.

Numerous molecular pathways are disrupted in MM, involving a plethora of affected factors and pathological consequences. For example, DNA damage repair (DDR) pathways are commonly disrupted in cancer and DNA repair inhibitors are intensively studied as chemotherapeutic agents. Unluckily, resistance to DNA damaging agents remains a common and critical problem. Using gene expression data of factors from the DNA repair pathways homologous recombination (HR), non-homologous end joining (NHEJ), and nucleotide excision repair (NER), we developed a DNA repair risk score to predict event-free and overall survival of NDMM patients [27]. This score offers a rationale to exploit DNA damaging agents, such as melphalan, doxorubicin or cyclophosphamide, in combination with DNA repair inhibitors as a therapeutic option. It could also provide further insight into the molecular mechanisms of resistance to DNA damaging drugs [32]. Similarly, a drug response prediction score based on GEP allows to predict sensitivity to bortezomib and melphalan in single treatment in high-risk patients [196]. More recently, we also identified several kinases involved in major signaling pathways that have prognostic value in MM, such as PBK, SRPK, CDC7-DDF4, MELK, CHK1, PLK4, and MPS1/TTK. Specific CHK1, MELK and PBK inhibitors decreased cell viability in HMCLs and primary myeloma cells and re-sensitized lenalidomide and melphalan resistant cell lines, suggesting a potential interest for kinases as therapeutic targets in combination treatments [197]. A recent study of 42 patients refractory to both PIs and IMiDs performed WES (*n* = 40) and RNA-seq (*n* = 27) to attempt to identify chemotherapy resistance mechanisms. It found that the number of subclonal mutations increased during the course of the treatment and p53 pathway was the most frequently mutated. The only mutational signature found in refractory patients was linked to exposure to alkylating agents, indicating that resistance to IMiDs and PIs is generally not due to mutation of their targets [198]. More recently, it was shown that PSMB6 and PSMB7 proteasome subunits, but not PSMB5, are essential for MM cell survival. Resistance to PI have been described with activating mutations or upregulation of PSMB5, PSMB6 and PSMB7 [199].

The application of these and other predictive scores (Table 4) at diagnosis and during the follow-up of MM patients would help clinicians to take better educated decisions regarding the best therapy for each patient. Moreover, low level of expression of certain genes related to therapeutic targets could explain low efficacy of the treatment in some cases, which suggests that RNA-seq tests could help to take better therapeutic decisions.

**Table 4.** Drug response-scores based on GEP

**Score**	**Genes**	**Datasets**	**Reference**
8-gene signature (dexamethasone/ thalidomide)	*ATF2*, *CCND2*, *CFLAR*, *DDX17*, *HSPA1A*, *RIT1*, *RNF148*, *WHSC1*	GSE16791	[169]
DM (DNMTi)	47 genes	E-MTAB-372 HMCLs	[200]
M^3^P (PIs and IMiDs)	47 genes in del17p	German MM study group (DSMM)	[177–179]
DRP (drug response prediction, melphalan and bortezomib)		GSE2658 (TT2/TT3a) GSE19784 (HOVON) GSE68871 (GIMEMAMMY-3006) GSE9782 (APEX)	[196]
IMiD-14	14 poor prognosis genes: *XPO1*, *DDR2*, *TRAF3IP3*, *FAIM3* 10 good prognosis genes: *IL5RA*, *TNFRSF7*, *AMPD1*, *ENO2*, *ITGA6*, *FLJ22531*, *LAMA5*, *PGRMC2*, *SLC39A14*, *KIAA0247*	GSE24080 GSE57317 GSE19784 (HOVON65/GMMG-HD4)	[201]
HA (HDACi)	37 genes	E-MTAB-372 GSE2658 E-TABM-937 and E-TABM-1088 (HMCLs)	[202]
DNMTi/HDACi (decitabine/quisinostat)	25/62 genes	E-MTAB-3178	[203]
VTD response (Bortezomib-Thalidomide-Dexamethasone)	5 genes: *ACTR2*, *BAI2*, *ANK3*, *GALNT5*, *GLT1D1*	GSE55145 GSE9872	[204]
DR (DNA repair, DNA damage agents)	17 bad prognostic 5 good prognostic	GSE24080 E-MTAB-372	[27]
EZ (EZH2 inhibitor)	15 genes	E-TABM-93 E-TABM-1088	[205]

Dormant myeloma cells in specific BM niches are thought to be responsible for MM relapse [206]. A single-cell RNA-seq study with mice found that these cells express a unique transcriptome signature enriched in immune and myeloma cell differentiation genes, that are switched off during reactivation to growing MM [207]. These data provide a rationale to design therapies to prevent relapse by targeting dormant myeloma cells. A small study compared the transcriptome of myeloma cells in the BM from 4 untreated MM patients and 4 healthy donors and evaluated differential transcript expression, mutations, new alternative splicing variants, and fusion genes. This analysis identified possible candidate genes associated with myeloma genesis, such as *EEF1G*, *ITM2C*, *FTL*, *CLPTM1L*, and *CYBA* [208]. Mesenchymal stem cells (MSCs) are essential components of the BM microenvironment with a role in MM. Affimetrix gene-expression microarrays allowed the identification of 3 genes (*COL4A1*, *NPR3* and *ITGBL1*) expressed in MSCs but little or not in PC. These data were used to build a score predictive of MM progression-free survival and SMM to MM progression, which reinforced the notion that the surrounding microenvironment has a critical impact on MM development [209]. Extramedullary progression of MM is associated to drug resistance and high mortality rate. Single-cell RNA-seq of BM and myelomatous pleural effusions or ascites from 15 patients showed activation of several pathways regulating proliferation, protein degradation, antigen presentation, glycolysis, and oxidative phosphorylation. Moreover, data suggested that myeloma cells possess immune evasion mechanisms, like upregulated inhibitory molecules for cytotoxic T and NK cells [210]. Transcriptomic profiling from a murine myeloma model found that bone morphogenetic protein (BMP) pathway, involved in bone formation and resorption, is upregulated in stromal progenitor cells, which makes it a potential new therapeutic target to treat myeloma-induced bone disease [211].

### Epigenomics

Epigenomics focuses on the analysis of epigenetic marks on chromatin, both on nucleic acids and histones, that alter gene expression without changing the DNA sequence. DNA methylation and histone modifications (acetylation, ubiquitination, methylation, and phosphorylation) are the epigenetic marks that drive cancer pathogenesis [212]. Consequently, drug epigenetic reprogramming as an anti-cancer strategy is an emerging field [213].

MM arises from malignant transformation within the B-cell lineage, which is tightly regulated by differentiation and proliferation mechanisms that rely on epigenetic modifications. Interestingly, more than half of MM patients have been found to carry mutations or CNAs on epigenetic enzyme genes [214]. An integrative study of gene expression, epigenetic profiling and *in situ* promoter capture Hi-C data for the 23 known loci associated with MM susceptibility identified disruption of developmental transcriptional regulators and altered B-cell differentiation as key features for MM risk [135]. Moreover, analysis of 2 available GSE datasets found more than 100 differentially expressed genes (51 upregulated and 78 downregulated) in MM patients compared to controls, mainly related to B-cell receptor, hematopoietic cell lineage, and NF-κB pathways [215].

Misregulation of epigenetic regulators is tightly related to cancer [216]. Methylation of DNA and histones is the most studied epigenetic modification. DNA hypermethylation is responsible for the inhibition of genes that control growth inhibition, apoptosis and cell differentiation, and can take place at particular loci. More precisely, in MM hypermethylation occurs outside CpG islands and is associated with intronic enhancer regions [217]. On the contrary, hypomethylation, which is associated with gene activation, is observed genome wide [218, 219]. Gene expression analysis of MM cells has identified obvious differences in methylation between normal cells and different myeloma stages. Indeed, methylation studies showed global DNA hypomethylation from MGUS to MM, hypermethylation of tumor suppressor genes in MM, and global hypermethylation in the transition from MM to plasma cell leukemia (PCL) [220, 221]. Furthermore, methylation analyses indicate that MM cells reacquire a methylation signature of undifferentiated cells that is associated to MM physiopathology [217]. Combination of DNA methylation and gene expression data identified 4 genes with tumor suppressor functions (*GPX3*, *RBP1*, *SPARC* and *TGFBI*) whose hypermethylation is associated with shorter overall survival [222]. These data suggest that methylation enzymes could be good therapeutic targets. Thus, DNA methyltransferase inhibitors (DNMTi), like decitabine, have been studied as potential myeloma treatments and a DNA methylation score based on 47 genes is available to predict the efficacy of decitabine [200]. Moreover, pre-clinical studies with human cells and murine models indicated that decitabine modulates Wnt/b-catenin signaling pathway and may increase myeloma cells sensitivity to bortezomib [223, 224], and that decitabine combination with the HDACi quisinostat may have anti-myeloma effect [203, 225]. Therefore, the study of DNA methylome in MM *vs.* normal PC can allow a better understanding of the mechanisms that control the progression of the disease through all its stages as well as the identification of new therapeutic targets/combinations.

Active DNA demethylation occurs mainly at enhancers and gene bodies. The first step of this process is the conversion of 5-methylcytosine (5mC) into 5-hydroxymethylcytosine (5hmC). Genome-wide 5hmC profiling of tumor PC has been recently performed. This analysis showed that proximity to a 5hmC-enriched region correlated with higher gene expression and indicated that 5hmC may participate in a myeloma-specific gene expression program. Moreover, *FAM72* was found to be a new prognosis gene involved in MM: 5hmC of *FAM72* enhances its expression and *FAM72* high expression is associated to resistance to bortezomib and sensitivity to HDACi/DNMTi [226]. 5hmC is associated with clinical aspects in purified MM cells from patients overlapping with active chromatin marks related to major on cogenes in MM [227].

In MM, poor prognosis t(4;14) TC results in upregulation of MMSET methyltransferase, and consequent global Lysine 36 of histone H3 (H3K36) hypermethylation and Lysine 27 (H3K27) low methylation [220, 228]. Mutations that truncate LSD1/KDM1A, a demethylase of Lysine 4 of histone H3 (H3K4) that regulates hematopoietic stem cell renewal, are associated to familial and early-onset MM. Missense KDM1A mutations are also frequent in patients without MM family history. These mutations correlate with an enrichment of Myc transcriptional targets [229]. Misregulation of histone demethylase KDM3A, which demethylates H3K9me1/2, is also involved in MM. It is upregulated in MM cells under hypoxia and its knockdown has anti-MM effect [219, 230, 231]. Another epigenetic regulator, KDM3B, is the most frequently mutated histone demethylase in MM patients [232]. Polycomb repressive complexes (PRC1 and PRC2) are major chromatin modifiers. PRC1 ubiquitinates H2AK119 and PRC2 trimethylates H3K27 to regulate the expression of important genes related to cell proliferation and differentiation [233]. The role of PRC complexes in MM has only been started to be deciphered. For example, we proved that inhibition of enhancer of zeste homolog 2 (EZH2), a PCR2 member, induces cell cycle arrest and apoptosis, and combination with lenalidomide has synergistic effect. Based on these data, we developed an “EZ score” to identify poor prognosis patients that would benefit from EZH2 inhibition, pointing at a new therapeutic target [205].

Methylation is not the only important epigenetic modification in MM. Using gene-expression data, we and others found that HDACi treatment mainly deregulated tumoral immunomodulatory pathways, which suggested an interest on combining HDACi and IMiDs [203]. In addition, we built scores to predict response to DNMTi, HDACi, or combination of both kinds of drugs [200, 202, 234].

Furthermore, recent studies started to decipher the chromatin regulatory network underlying pathophysiology of MM cells. The first genome-wide profiling of H3K27me3 and H3K4me3 found a set of genes unique to primary MM cells, some with H3K27me3 alone and others with both epigenetic marks. Increased silencing of H3K27me3 target genes was found at advanced MM stages and correlated with poor patient survival. Moreover, EZH2 inhibition was shown to have antimyeloma effects and induce cell apoptosis [235]. Another study mapped regulatory elements, open chromatin, and transcription factor footprints in primary MM cells and compared them to normal cells. The authors found that MM cells present changes in enhancer activity connected to deregulation of transcription factor genes, which leads to aberrant expression of genes involved in proliferation, survival, signaling, adhesion, and DNA methylation. This study also reported widespread decompaction of heterochromatin associated with increased activity of regulatory elements in MM cells [236]. The extensive activation of regulatory elements has been recently confirmed, and linked to upregulation of several signaling pathways, including NF-κB, p53, Notch, and mTOR [237].

In light of all these discoveries, it is not surprising that epigenetic drugs are getting increasing attention as potential MM therapy. The use of sequencing and gene expression profiles to study the role of epigenetic mutations and modifications in MM and normal B cell differentiation, and epigenetic drugs as a strategy to treat MM have been reviewed by others [219, 238].

### Proteomics

Proteomics methods allow identification and quantification of proteins or peptides from complex mixtures and purified/enriched samples. Large-scale analysis of proteins provides further information about cell composition both in basal conditions and in response to drugs, and is tightly related to the rest of the omics approaches.

MM is commonly characterized by aberrant secretion of a monoclonal Ig or a fragment of it, known as M-protein or paraprotein [239]. Detection of Ig light chains by electrophoresis in serum or urine from patients is an easy and well-established method to monitor aberrant proteins secreted by PCs for diagnostic purposes [240, 241]. Serum/urine monoclonal Ig detection is used in combination with immunofixation electrophoresis and serum free light-chain assay to diagnose monoclonal gammopathies [242, 243]. M-protein levels are usually elevated at diagnosis and drop upon treatment, serving as a therapy response readout. Changes on free light chain secretion can reflect a clonal evolution linked to proliferation of a new dominant clone, which can have important consequences for therapeutic decisions [244–246]. It has been proposed that monitoring clone-specific peptides is more sensitive and specific than other current analytical methods to detect Igs [247].

Despite the broad use of these techniques in MM diagnosis and monitoring, more powerful proteomics approaches have been proposed. For example, quantitative mass spectrometry (MS) has been used to quantify the serum levels of Igs along the progression of MM, showing increased sensitivity compared to standard clinical methods [248]. However, this method has not been incorporated in clinical practice so far. In addition, the same study coupled proteomics and RNA-sequencing as a strategy for personalized detection of myeloma tumor burden with higher performance [248]. Detection of Ig variable regions from patient sera by MS has recently been proposed as a highly efficient, specific and non-invasive way to assess MRD [249].

M-protein monitoring is not the only way to characterize MM progression by proteomics. Targeting particular groups of proteins or enzymes and their targets provides information about MM molecular mechanisms and drug mechanisms of action. Deregulated activity of several kinases in MM cells, such as ERK, JUNK, STAT, MAPK, and AKT, indicates that phosphorylation pathways could be important for the disease physiopathology and clonal expansion [250–253]. Unbiased phosphoproteomics found differential activation of kinases that was linked to *RAS* mutations in different HMCLs, which allowed to build a predictive score linking pharmacologic and genetic kinase dependencies in MM [254]. Stable isotope labeling with amino acids in cell culture (SILAC) and liquid chromatography mass spectrometry (LC-MS)/MS analyses of primary MM cells treated with bortezomib identified novel bortezomib-induced phosphorylation sites, mostly in nucleic acid binding proteins, like splicing and translation factors [255]. Moreover, increased phosphorylation of stathmin, a microtubule destabilizer, was reported to play a role in mediating apoptosis upon bortezomib treatment [255]. Proteomics has helped dissecting the apoptosis pathway induced by dexamethasone and its resistance mechanisms in MM [256]. In 2018, the first public resource of phosphorylations and histone modifications induced by treatment with 90 drugs in 6 HMCLs was published, providing a tool for new therapeutic opportunities [257].

For a long time, proteomics has been used to elucidate drug resistance mechanisms in MM. For example, resistance to melphalan in HMCLs has been studied by protein fractionation and liquid chromatography coupled to multiple reaction monitoring (LC-MRM) [258], global proteomic coupled to transcriptomics [259], and proteomics coupled to metabolomics [260]. Melphalan resistance seems to depend on alterations on NF-κB pathway, unbalance of pro-apoptotic, anti-apoptotic signals and DDR factors [258], and metabolic and oxidative stress response pathways [259].

Resistance to bortezomib-based treatments in RRMM patients correlates with accumulation of proteasome subunits and/or proteins involved in the response to oxidative stress and cell redox homeostasis, and misregulation of apoptosis and programmed cell death factors [261]. A correlation between serine synthesis and bortezomib resistance has also been reported [262]. Proteomics analysis of PI-resistant MM cells has shown that adaptation to bortezomib and carfilzomib involves quantitative changes in more than 600 proteins, including downregulation of apoptosis factors, transcription and translation factors, as well as upregulation of metabolism, homeostasis and protein folding and destruction proteins, with MDR1 as the most upregulated protein in carfilzomib-resistant cells [263]. Quantitative proteomics of patients treated with bortezomib-liposomal doxorubicine-dexamethasone or lenalidomide-bortezomib-dexamethasone identified candidate predictors of favorable response to bortezomib, DNA damaging agents, and IMiDs [264].

Upon dexamethasone treatment of MM.1S cell line, global protein expression analysis by two-dimensional polyacrylamide gel electrophoresis (2DPAGE) identified upregulation of proteins involved in post-translational modifications, protein folding and trafficking, and downregulation of proteins involved in cell survival and proliferation. It also suggested that inability to induce FKBP5, a member of the steroid receptor complex, may be related to dexamethasone resistance [256]. The proteomic profile of PCs from NDMM and healthy donors was analyzed by two-dimensional gel electrophoresis (2-DE). Several differentially expressed proteins were identified, including annexin A1. Subsequent functional studies showed that knockdown of annexin A1 potentiated dexamethasone effects insensitive and resistant HMCLs [265].

The effect of thalidomide-based therapy on 39 newly diagnosed responder (*n* = 22) and non-responder (*n* = 17) MM patients was analyzed by immunodepletion, two-dimensional difference gel electrophoresis (2-D DIGE) analysis and MS. The best candidate misregulated proteins found in non-responders (ZAG, VDB, SAA, B2M, and Hp) were validated by enzyme-linked immunosorbent assay (ELISA) in a new cohort of patients, helping to establish a thalidomide-response predictive score based on protein quantification [266].

Even the effect of ASCT on serum proteome has been studied by differential scanning calorimetry, a biophysical technique to characterize the stability and conformation of biomolecules in solution, showing that changes in serum paraprotein level upon transplantation can be detected by this minimally invasive method [267].

In the search for new biomarkers, analyzing proteins from serum is a less invasive alternative than performing proteomics from purified BM PCs. For example, proteomic profiling of sera from MM patients that have developed resistance to the triple combination cyclophosphamide-thalidomide-dexamethasone has aimed to identify biomarkers that could predict the response to the therapy. In this study, patient sera were collected before treatment with bortezomib-thalidomide-dexamethasone or bortezomib-doxorubicin-dexamethasone. Subsequently, patient responses to therapy were correlated with their initial protein profiling, identifying 54 proteins that could be potential biomarkers [268].

SILAC allows proteins quantification and relative abundance determination, but its application is limited to proliferating cells with active protein synthesis. Super-SILAC is a variant of this method that overcomes this problem (for more details see [269]), which makes it useful for secondary plasma cell leukemia (sPCL) studies, the last and most aggressive form of MM. sPCL is poorly studied from a molecular point of view because it is a very rare condition and patients present a short survival [270]. In 2017, the first super-SILAC quantitative proteomics study of one patient at both MM and sPCL stages showed almost 800 differentially expressed proteins, revealing a shift in cell metabolism towards aerobic glycolysis (known as “Warburg effect”) in the progression of the disease, which suggests that glycolysis enzymes could be potential therapeutic targets in the treatment of sPCL [271]. Further studies with more patients would be beneficial for the understanding of MM to sPCL progression.

The “Warburg effect” is the switch from mitochondrial oxidative phosphorylation to aerobic glycolysis to generate ATP in many cancer cells, even when oxygen is available. This phenomenon is regulated by several oncogenes, like *MYC*, and implies the upregulation of glycolytic pathway enzymes [272–274]. Interestingly, some of the above-mentioned proteomics studies [259, 271], as well as other metabolic and molecular studies [275–277], point at the “Warburg effect” association with drug resistance mechanisms in MM. These data support a gene expression analysis that showed a positive correlation between high levels of CD147 and glycolytic enzymes involved in the “Warburg effect”, which was associated to poor prognosis in MM [278]. Thus, aerobic glycolysis factors are interesting therapeutic targets in drug resistant patients which need further study.

A primary study of serum peptide profiles by matrix-assisted laser desorption ionization time-of-flight mass spectrometry (MALDI-TOF MS) and Clinprot bioinformatics analysis allowed the identification of four peptides in NDMM patients that were used to develop a preliminary diagnostic model with potential interest for early diagnosis [279]. Based on these findings, the same researchers aimed to validate their model by studying the soluble components of the BM niche, which change with the evolution of the disease. They confirmed that the identified four circulating peptides (dihydropyrimidinase-like 2, fibrinogen alpha chain, platelet factor 4 and alpha-fetoprotein) may have biomarker value in MM diagnosis, remission and relapse [280].

Currently, it is well established that BM microenvironment has a central role in MM progression and drug resistance. It contributes to the physiopathology of the disease by secreting molecules important for cell proliferation and adhesion, and by releasing extracellular vesicles (EVs) [281]. Regarding this aspect of MM, proteomics approaches have been used to study the remodeling of the extracellular matrix protein composition from MGUS to MM, providing more insight into the mechanisms of development of a permissive BM microenvironment, and identifying *LGALS1* and *ANXA2* as biomarkers for MM overall survival [282]. Moreover, a LC-MS/MS study of EVs composition extracted from MM cell lines and patient sera found that EVs with high levels of CD44, an important molecule for cell adhesion and BM secretion of interleukin (IL)-6, could be related to reduced overall survival [283].

Due to the difficulty of primary malignant PCs obtention and culture, most of the reported proteomics studies in MM focus on serum profiling or HMCLs models. However, some effort has been done also on analysis of patient cells. For example, ion intensity-based label-free quantitative MS identified a panel of 9 upregulated, and 9 downregulated proteins in PCs isolated from the BM of MM patients when compared to healthy donors [284].

The importance of proteomics for precision medicine in MM has been recently reviewed elsewhere [285].

### Metabolomics

Altered metabolism is one of the hallmarks of cancer [286]. Metabolites are the substrate, intermediate or final products of metabolism, such as amino acids, proteins, lipids or sugars, which exert numerous cellular functions, and are a direct readout of the organism physiology at a given moment. It is now well established that metabolism adaptation provides notorious advantages to cancer cells and that metabolites are responsible for phenotypic manifestations of cancer progression. The study of metabolites, known as metabolomics, allows the identification of diagnostic biomarkers to assess patient risk, disease progression, and predict the response to treatment, as well as the discovery of potential new druggable targets.

Metabolomics approaches can be untargeted, when as many metabolites as possible are measured from the samples, or targeted, when a particular set of metabolites is measured to answer a specific question. Therapeutic drugs are metabolized by the organism and, in general, the end molecules can be detected in a non-invasive way from biological fluids, like blood, saliva or urine, providing huge amounts of valuable information for drug safety and toxicologic screens. In general, metabolites are identified by MS-based technologies, but metabolomics sample processing and data analysis are not well standardized yet and their improvement remains a challenge for the near future [287]. Since metabolic differences between patients can be partially dependent on age, gender and body mass index, performance of metabolomics studies should be carefully designed to use controlled cohorts and implement unbiased analysis methods to avoid misleading biased data.

#### Lipidomics

Lipids are not only crucial components of cell membranes and energy reservoirs, they also play important roles in physiological processes and participate in signaling pathways. In the past decades, recent evidence has accumulated for the role of lipid metabolism in cancer onset and progression [288–290]. Of note, obesity is a risk factor for many cancers, including MM [291–294], which implies that diet and metabolism alterations should be taken in account when designing personalized treatments. Furthermore, targeting enzymes involved in fatty acid synthesis and modification reduces MM cell proliferation [295, 296]. Hence, the study of MM lipidome emerges as a relevant source of potential biomarkers that could help predict the evolution of the disease and the response to treatment.

Adiponectin, a cytokine produced by adipocytes that regulates glucose and fatty acid oxidation, is paradoxically decreased in obesity. Addition of adiponectin to HMCLs triggers apoptosis, and supplementation with palmitic acid partially rescues its effect [296]. On the contrary, another study reported that addition of palmitic acid to culture medium of HMCLs reduced cell viability and BM MM PCs had decreased levels of palmitic acid when compared to healthy PCs by time-of-flight secondary ion mass spectrometry (TOF-SIMS) [297]. The apparent contradiction between these data may be due to the fact that Medina and collaborators [296] deregulated fatty acid metabolism by the addition of adiponectin prior to palmitic acid supplementation, whereas Nagata and collaborators [297] analyzed the impact of palmitic acid addition alone. In any case, their results suggest that palmitic acid metabolism could be a potential therapeutic candidate in MM either by diet surveillance or as a druggable target and should be further explored.

Gas chromatography analysis pointed at a lower n-3/n-6 polyunsaturated fatty acid (PUFA) ratio in the composition of the membrane of erythrocytes in MM patients than in controls [298]. The same approach was used to study lipids from plasma samples. The study showed increased levels of saturated and n-6 PUFA in MM patients compared to healthy controls, probably associated to a pro-inflammatory effect in the BM microenvironment, and therefore to survival and proliferation of cancer cells [299]. Lipidomic profile of BM plasma found that lipid composition varies from MGUS to MM, more specifically, complex lipids such as phosphatidylethanolamines, phosphatidylinositols, and lactosylceramides were decreased in BM MM compared to MGUS [300].

Purification of proteins and lipids from PCs from 7 MM patients and their subsequent analysis by LC-MS has recently been reported. This small study used targeted and untargeted lipidomics to detect a significant downregulation of phosphatidylcholines, ceramides and lysophosphatidylethanolamines, and upregulation of phosphatidylethanolamines, sphingomyelines and sphingosines in RRMM compared to NDMM [301].

Combination of lipidomics, metabolomics, and phosphoproteomics identified almost 12,000 phosphorylation sites, primarily associated to the BCR-ABL-ERK pathway, in the HMCL H929, which contains a rare BCR-ABL fusion. Treatment with imatinib, an ABL kinase inhibitor that is the standard-of-care drug for BCR-ABL mutations, reduced the phosphorylations associated to this pathway and increased the phosphorylation of proteins associated to RNA expression. Imatinib also reduced lipid biosynthesis and fatty acid incorporation. Together, the data obtained through this triomics approach indicated that kinase inhibitors not only affect proteins, but also metabolic events of other cellular components [302].

Recently, serum lipidomics by ultraperformance liquid chromatography (UPLC)-time-of-flight mass spectrometry (TOFMS) and UPLC-MS/MS was performed to find new candidate biomarkers of response to bortezomib and for the risk of bortezomib-induced peripheral neuropathy, a common side effect. Samples from MM patients were collected prior to their treatment with bortezomib plus low dose dexamethasone. Three hundred and eighty five lipids were detected in the sera and classified into 4 groups, determining that low levels of some glycerophospholipids, sphingolipids, and cholesteryl esters correlated with poor response to bortezomib, whereas altered levels of several lysophosphatidylcholines, ceramides, phosphatidylcholines, oxidative fatty acids, and neutral lipids were found in patients suffering of bortezomib-induced peripheral neuropathy [303]. Another study in plasma also has found an imbalance in sphingolipid metabolism (increased ceramides and decreased sphingomyelin levels) due to upregulation of the enzyme acid sphingomyelinase (ASM). This imbalance is related to resistance to melphalan and bortezomib, suggesting a potential interest of ASM, which is crucial for autophagy in cancer [304], as a therapeutic target in MM [305]. These data coincide with the recently highlighted link of sphingolipids with human diseases [306]. Interestingly, gene expression analysis showed that a subgroup of patients with MMSET and ASM overexpression presented an ultra-high-risk profile [305].

#### Other metabolites

One of the first metabolic studies in the context of MM analyzed the metabolic profile of the BM microenvironment using filtered plasma derived from BM aspirates. This analysis identified metabolite differences between healthy donors, MGUS and MM patients. For example, in MGUS and MM BM isoleucine and threonine levels were decreased, creatine export was decreased, urea production was increased, numerous lipid metabolism-related metabolites presented altered levels, and the niche oxidative metabolism was increased [307], which correlates with the augmented oxidative stress previously reported in MM [308]. Of note, these metabolic changes seem to occur mostly in MGUS development rather than in the progression to MM [307]. In the same line, it has been reported that the metabolomic plasma profile of MGUS and NDMM/RRMM patients significantly differs between them and from that of healthy controls, specifically in amino acid, lipid, and energy related pathways [309].

Metabolomics techniques are also used to characterize the drug resistance mechanisms in MM. A preliminary study also reported significantly different metabolic profiles between bortezomib-resistant and sensitive HMCLs [310], identifying potential biomarkers for bortezomib resistance to be validated in future studies. Hypoxia in the tumor microenvironment alters tumor metabolism, commonly leading cancer cells to perform aerobic glycolysis: the “Warburg effect” described above [311]. Using patient primary cells, HMCLs and tumor cell injection in severe combined immunodeficient (SCID) mice, it was shown that lactate deshydrogenase A (LDHA) and the transcription factor hypoxia-inducible factor 1A (HIF1A) induce hypoxia-driven resistance to bortezomib, which positions them as potential therapeutic targets for bortezomib-resistant MM patients [277]. Importantly, this study suggested that the oxygen conditions (normoxia or hypoxia) in different niches of the BM can be related to PC subpopulations resistance to drugs, with those cells under normoxia being sensitive to bortezomib while those under hypoxia being resistant [277]. Therefore, oxygen conditions may be tightly related to drug resistance development and relapse.

Moreover, as other cancer cells, MM cells have been shown to rely on glucose and glutamine, and withdrawal of either of them induces variable levels of apoptosis [312–316]. Cells who survive under glutamine deprivation conditions present sustained expression of the anti-apoptotic protein MCL-1, which has been related to bortezomib-resistant phenotype [315], linking glutamine metabolism to drug resistance [317]. In line with these results, the metabolic profile of bortezomib-resistant HMCLs and primary MM cells showed increased serine synthesis pathway activity, with a strong correlation between this pathway activity and bortezomib concentration resistance [262]. Resistance to other drugs has also been assessed by multiomics methods. For example, the combination of proteomics and metabolomics has been used to analyze the bases of melphalan resistance in MM, providing a set of candidate metabolism-related biomarkers to be tested [260].

Proteometabolomics studies in PI-resistant HMCLs showed changes in the expression of metabolic factors, such as nicotinamide adenine dinucleotide phosphate (NADPH) dehydrogenase, malate dehydrogenase, and fatty acid synthesis enzymes, suggesting that these cells have gone through metabolic adaptation, which opens a therapeutic window to overcome drug resistance [263].

Another study performed non-targeted metabolomics analysis on serum samples from patients with active MM (presenting CRAB symptoms, whose sera were collected before treatment) and bortezomib-responding MM (decrease in CRAB symptoms and more than 50% reduction in M protein after four treatment cycles, whose sera was collected 21 days after the 4th cycle), and age-matched healthy donors. Using nanoflow ultraperformance LC coupled to high-resolution orbitrap MS, carnitine and acetylcarnitine were identified as potential biomarkers for NDMM and relapsed MM [318]. In addition, twenty-three metabolites that significantly differed between groups with potential as biomarkers for active MM were identified, mostly involved in arginine and proline metabolism, and glycerophospholipid metabolism, such as phosphatidylcholines, creatinine and asymmetric dimethylarginine [319]. These results agreed with a previous report of increased arginine levels in NDMM, which also identified altered levels of other metabolites at diagnosis but not after remission [320]. In contrast, another work using quadrupole time-of-flight LC-MS on a small patient sample reported no change in arginine and proline metabolism but in bile secretion, pyrimidine and carbon metabolic pathways compared to healthy controls [321].

Finally, metabolomics has also been combined with histomorphology for the targeted study of osteolytic lesions in MM. The first proof-of-concept study in this matter showed the possibility to correlate histopathologic manifestations with metabolic alterations, aiming to decipher the role of the interaction of MM cells with BM microenvironment in the bone disease [322].

The application of metabolomics to hematologic malignancies has been reviewed elsewhere [323].

## Conclusions

In the past decades, extensive *in vitro* and *in vivo* studies [19, 324] have been instrumental in the development of new drugs and combination therapies for MM, greatly improving patient survival. However, MM remains virtually incurable, with drug resistance and relapse being the most important problems.

MM is an extremely complex disease whose origin and inheritability are just starting to be understood. In past years, genomics and transcriptomics data have greatly contributed to identify new candidate driver mutations, understand genetic and epigenetic characteristics of progression from pre-symptomatic to advanced MM stages, how treatments affect clonal evolution of the disease, and their role in the relapse and resistance to drugs. Indeed, the discovery of clonality and clonal evolution has made clear that determining the particular genetic burden of each patient’s clonal populations is key to decide the best treatment. Targeted therapies that do not take in account tumor clonality can have the paradoxical effect of selecting a resistant clone that eventually will cause patient relapse. Therefore, precision is crucial in this matter.

**Figure 1.**
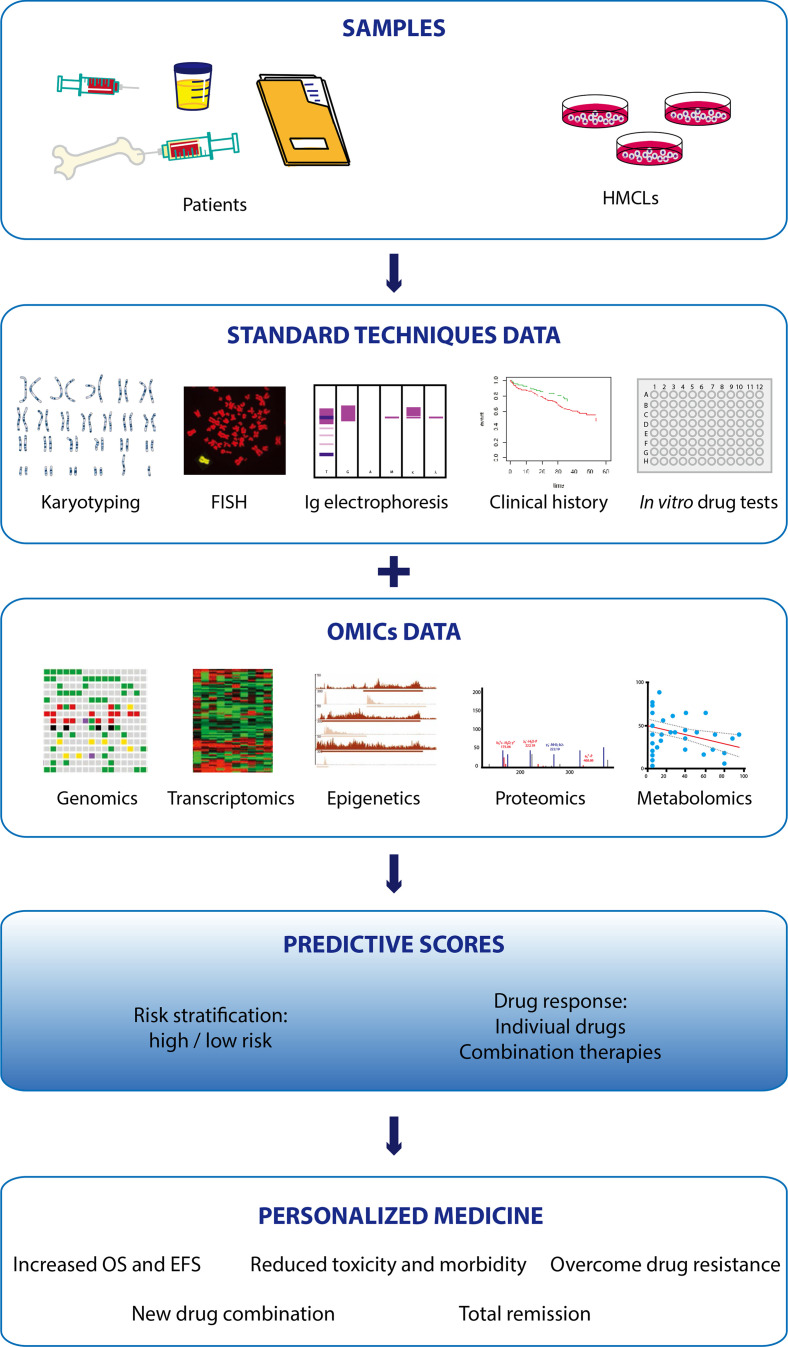
Predictive scores in MM. Samples from patients (blood, urine, BM aspirates) and HMCLs are commonly studied by classical diagnosis techniques. Karyotyping and fluorescence *in situ* hybridization allow the detection of CNA and translocations, respectively; electrophoresis detects Ig chains in serum and urine from patients; clinical history collects data about lines of treatment, relapse, progression free survival, overall survival, MRD, and co-morbidities, that are crucial when analyzing cohorts of patients. Primary cells from patients and HMCLs can be used to assess the efficacy of *in vitro* drug treatments and drug combinations in order to find new therapeutic approaches. The integration of data from these routine techniques with omics obtained data, specially from genomics and transcriptomics studies, allows to build scores (Tables 3 and 4) capable to stratify the risk or predict drug response, that are instrumental for personalized treatment. Combination of several scores will refined diagnosis and improve the monitoring of the evolution of the disease. Finally, since drug resistance is the main reason of relapse, personalized medicine based on omics-developed scores will allow to choose the best drug for each patient, increasing the probability of survival while reducing treatment-associated toxicity, which also translates in better quality of life. It includes a karyotyping cartoon (Karyotyping) taken from an open source: https://smart.servier.com/smart_image/karyotype/

For a long time, the most important clinical challenge has been precise risk stratification at the time of diagnosis, because patients significantly differ in evolution, survival and response to treatments. Thus, the only way to accurately classify them and decide the best treatment for each particular case is by analyzing molecular signatures that can predict their response to drugs. Genomics and transcriptomics approaches have opened a new era in this regard, allowing a molecular classification of MM that was impossible with classical cytogenetics techniques, like karyotyping or FISH. Importantly, given that omics techniques are costly and labor intensive, there is a need for the implementation of standard procedures regarding sample collection, storage, processing, and analysis, making studies more comparable and allowing more robust conclusions. Thanks to omics data, specially genomics and transcriptomics, numerous predictive scores are now available to assess the complex molecular heterogeneity of MM and have provided clinicians with powerful decision tools (Figure 1). Identifying all clonal populations at diagnosis and designing therapies targeting all of them with a combination of drugs is the best way, maybe the only one, to avoid resistance and achieve long-term remission. Another important challenge is to accurately detect MRD in order to prevent relapse. The development of more sensitive technologies will be essential for this in the future.

Myeloma cells, which are the substrate for genomics and transcriptomics analyses, are rare cells, and their collection may require invasive clinical interventions. Therefore, the quantity of primary cells available for these studies is usually very limited. *In vitro* works with HMCLs have helped to circumvent this problem, at least partially, allowing the development of predictive scores subsequently validated in patient cohorts. Moreover, recent data have highlighted the importance of other cell types, like BM cells or adipocytes [325–327], in the evolution of MM and their role in resistance to drugs. Therefore, the roles of microenvironment cells in treatment failure should be more carefully studied and integrated in therapy decisions. In addition, non-PC biomarkers can provide new therapeutic targets that help to overcome resistance and potentiate the effect of standard-of-care drugs.

Although omics approaches other than genomics and transcriptomics are less established in the clinical practice, they can also provide important information for the identification of diagnostic biomarkers and new therapeutic targets. The pleiotropic effect of most drugs generates changes in protein levels, regulation of molecular pathways, and generation of final metabolites. All these changes can be easily monitored using patient serum or urine by proteomics and metabolomics techniques with minimally invasive procedures. Thus, it is desirable that data coming from these approaches will be systematically integrated in the design of clinical trials in the near future.

**Figure 2.**
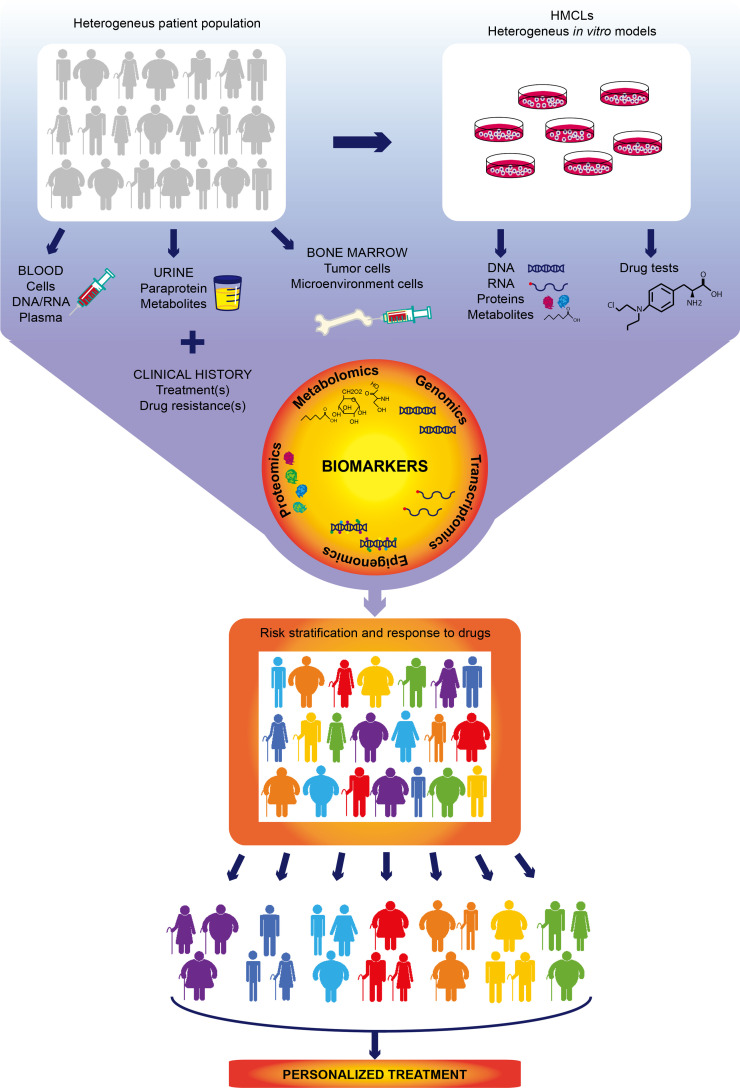
Omics approaches for the development of personalized treatments in MM. Cells and biological molecules are obtained from blood, urine and BM samples from heterogeneous populations of MM patients. HMCLs that reflect MM heterogeneity derived from patientsprovide a tool to study drug responses *in vitro* and are a source of biological molecules for subsequent studies. Analyses of patient and HMCLs samples by omics approaches lead to the identification of biomarkers that allowrisk stratification and predict response to drugs

In conclusion, the combination of multiple omics methods, which is commonly known as “multiomics”, is crucial to understand the origin of MM and its clonal evolution, dissect the mechanisms of action and resistance to drugs, detect MRD, and prevent relapse. The most promising perspective against MM is the implementation of multiomics analyses in the clinical practice to develop fully personalized therapies (Figure 2) that will not only extend the life of the patients, but hopefully lead them to a complete and long-lasting remission.

## Abbreviations

5hmC: 5-hydroxymethylcytosine
ADCs: antibodies drug conjugates
ASCT: autologous stem cell transplantation
ASM: sphingomyelinase
BM: bone marrow
CNA: copy number aberration
CRBN: cereblon
DARA: daratumumab
DDR: DNA damage repair
DNMTis: DNA methyltransferase inhibitors
EVs: extracellular vesicles
FISH: fluorescence *in situ* hybridization
GCs: glucocorticoids
GEP: gene expression profiling
HDAC: histone deacetylases
HDACis: histone deacetylase inhibitors
HMCLs: human MM cell lines
Ig: immunoglobulin
IMiDs: immunomodulatory drugs
LC-MS: liquid chromatography mass spectrometry
LRP: lung resistance protein
mAbs: monoclonal antibodies
MDR: multi-drug resistance
MGUS: monoclonal gammopathy of undetermined significance
MM: multiple myeloma
MRD: minimal residual disease
NDMM: newly diagnosed MM
NER: nucleotide excision repair
NF-κB: nuclear factor kappa B
NGS: next generation sequencing
PC: plasma cell
P-gp: P-glycoprotein
PIs: proteasome inhibitors
PRC: polycomb repressive complex
PSMB5: proteasome subunit beta type-5
RRMM: relapse or refractory MM
SILAC: stable isotope labeling with amino acids in cell culture
SMM: smoldering MM
SNP: single nucleotide polymorphism
sPCL: secondary plasma cell leukemia
TC: translocation
TPD: target pulldown
WES: whole-exome sequencing
WGS: whole genome sequencing
XPO1: exportin 1

## Declarations

### Copyright

© The Author(s) 2021.
